# Aluminum Diethylphosphinate-Incorporated Flame-Retardant Polyacrylonitrile Separators for Safety of Lithium-Ion Batteries

**DOI:** 10.3390/polym14091649

**Published:** 2022-04-19

**Authors:** Seok Hyeon Kang, Hwan Yeop Jeong, Tae Ho Kim, Jang Yong Lee, Sung Kwon Hong, Young Taik Hong, Jaewon Choi, Soonyong So, Sang Jun Yoon, Duk Man Yu

**Affiliations:** 1 Energy Materials Research Center, Korea Research Institute of Chemical Technology (KRICT), Daejeon 34114, Korea; mlc1207@krict.re.kr (S.H.K.); hwanyeop@krict.re.kr (H.Y.J.); thkim@krict.re.kr (T.H.K.); ljylee@krict.re.kr (J.Y.L.); ythong@krict.re.kr (Y.T.H.); 2 Department of Polymer Engineering, Chungnam National University, Daejeon 34134, Korea; skhong@cnu.ac.kr; 3 Department of Polymer Science and Engineering, Kyungpook National University, Daegu 41566, Korea; jwchoi@knu.ac.kr

**Keywords:** polyacrylonitrile separator, phosphinate, heat resistance, flame retardant, lithium-ion battery

## Abstract

Herein, we developed polyacrylonitrile (PAN)-based nanoporous composite membranes incorporating aluminum diethylphosphinate (ADEP) for use as a heat-resistant and flame-retardant separator in high-performance and safe lithium-ion batteries (LIBs). ADEP is phosphorus-rich, thermally stable, and flame retardant, and it can effectively suppress the combustibility of PAN nanofibers. Nanofibrous membranes were obtained by electrospinning, and the content of ADEP varied from 0 to 20 wt%. From the vertical burning test, it was demonstrated that the flame retardancy of the composite membranes was enhanced when more than 5 wt% of ADEP was added to PAN, potentially increasing the safety level of LIBs. Moreover, the composite membrane showed higher ionic conductivity and electrolyte uptake (0.83 mS/cm and 137%) compared to those of commercial polypropylene (PP) membranes (Celgard 2400: 0.65 mS/cm and 63%), resulting from interconnected pores and the polar chemical composition in the composite membranes. In terms of battery performance, the composite membrane showed highly stable electrochemical and heat-resistant properties, including superior discharge capacity when compared to Celgard 2400, indicating that the PAN/ADEP composite membrane has the potential to be used as a heat-resistant and flame-retardant separator for safe and high-power LIBs.

## 1. Introduction

A lithium-ion battery (LIB) is a secondary battery that has high energy density and power output and is used in various applications such as smartphones, drones, and electric tools [1,2,3]. Recently, the rapidly increasing demand for electric vehicles and large-scale energy storage systems (ESSs) has greatly influenced the development of high-performance and long-life LIBs [4]. However, the safety of LIBs is an issue when their energy density increases, because a large release of electrochemical energy can lead to an explosion and fire arising from the inappropriate operation of LIBs [5,6]. In particular, the explosion and fire of electric vehicles or ESSs cause devastating accidents and casualties, making solving the safety issues in the next-generation LIBs essential. The safety issues of LIBs mainly originate from an internal short-circuit failure due to the breakage of the separator by lithium dendrites or defects in the separator itself [7,8]. The separator is located between the anode and cathode and is a key component of safe LIBs; it acts as a porous membrane, preventing short-circuit failures [9,10,11]. Therefore, it is necessary that a separator has high-dimensional stability, does not shrink at elevated temperatures, and has uniform nanosized pores that serve as lithium-ion transport channels [12]. Commercial polyolefin separators, such as polyethylene (PE) and polypropylene (PP) membranes, have the advantages of excellent mechanical strength, chemical stability, and low cost; however, they have significant drawbacks such as low porosity, poor thermal dimensional stability, incompatibility with liquid electrolytes (LEs), and high flammability. These drawbacks limit the commercial applications of these separators in high-performance LIBs [13].

Several studies have reported membrane materials that can overcome the limitations of polyolefin membranes. One of the approaches to improve LIB safety is to fabricate a membrane using a heat-resistant polymer that has excellent thermal and mechanical properties. For example, Hao et al. [14] prepared polyethylene terephthalate (PET)-based porous membranes by electrospinning, and they showed good thermal stability at temperatures as high as 250 °C. Additionally, the PET membrane improved the battery performance and charge−discharge cycle stability compared to the polyolefin membrane, owing to its high porosity and three-dimensional porous structure. Li et al. [15] reported polybenzimidazole (PBI)-based membranes fabricated by a water vapor phase inversion method that were thermally stable at temperatures up to 400 °C. A spongy-like porous structure was formed in the PBI membrane, enabling high electrolyte uptake and battery performance. Kim et al. [16] fabricated polyphenylene sulfide (PPS)-based porous membranes using PPS/SiO_2_ composites treated by a plasma-assisted mechanochemical process (MP) for the homogeneous distribution of SiO_2_ nanoparticles. The highly porous structure of the PPS membrane was obtained by removing SiO_2_ through etching. The MP-treated PPS membranes did not undergo any dimensional deformation at temperatures up to 250 °C and were stably protected from lithium dendrites for 400 h at a current of 1.0 mA/cm^2^.

**Scheme 1:**
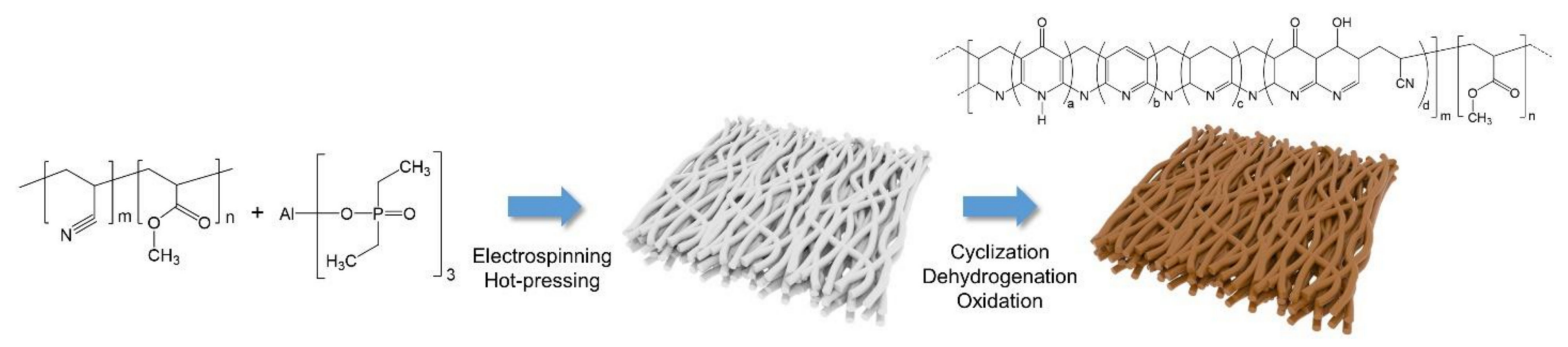
Schematic illustration of the fabrication of the PAN/ADEP composite membranes by electrospinning.

Polyacrylonitrile (PAN)-based membranes have been widely studied as LIB separators, because of their attractive properties such as good thermal and chemical stability and robust mechanical strength. In addition, the nitrile groups in PAN enhance compatibility with LE, achieving superior ion conductivity. Cho et al. [17] studied porous PAN membranes with thicknesses and pore sizes similar to those of the polyolefin membranes. The prepared PAN membranes showed higher discharge capacity and cycle stability compared to those of the polyolefin membranes because of the smaller ion diffusion resistance arising from the good compatibility with LEs. Ma et al. [18] prepared nine types of electrospun PAN membranes by varying the concentrations of the solution and hot-pressing pressure, and the effects of fiber diameter and membrane porosity on electrolyte uptake and ion transport through the membrane were observed. The results demonstrated that the electrochemical performance of the PAN membrane with small fiber diameters and hot-pressed under high pressure was superior to that of all other membranes. Lee et al. [19] reported partially oxidized PAN-based membranes by heat treatment at 230 °C. The mechanical strength and heat resistance of PAN were significantly improved by partial oxidation and cyclization of PAN chains (Scheme 1) [20], and ~50 MPa of tensile strength with no shrinkage of the PAN membrane at ≤180 °C was observed. The high discharge capacity and stable cycle performance were also shown in the PAN membrane due to the fact of good LE wettability and ionic conductivity.

The studies above show an improvement in LIB safety using heat-resistant membranes. Nevertheless, the risk of membrane shrinkage remains, particularly in cases of direct fire, local heat spot generation, and severe battery penetration [21]. The introduction of flame retardants into membrane materials is an effective way of preventing fire accidents from short-circuit failure [22,23]. Yusuf et al. [24] introduced 9,10-dihydro-9-oxa-10-phosphaphenanthrene-10-oxide (DOPO) as a flame retardant into PAN for flame-resistant PAN/DOPO composite membranes by electrospinning, since PAN decomposes into acrylonitrile, ammonia, and other nitrile compounds that are combustible when exposed to heat for a long period. The PAN/DOPO composite membrane reduced the peak heat-release rate by half compared to that of the PAN membrane, verifying that the phosphorus flame retardant effectively quenched the radicals released during combustion.

Herein, we fabricated a PAN-based composite membrane incorporating aluminum diethylphosphinate (ADEP) by electrospinning and heat-treated it for partial oxidation. ADEP is a highly thermally stable phosphorus-rich flame retardant that can effectively suppress the combustibility of heat-resistant polymer materials [25,26]. Generally, phosphorus-based compounds exert flame retardancy in the gas and solid phases. In the gas phase, the PO• radical produced by ADEP, as a scavenger, eliminates H• and OH• radicals, which are generated upon the thermal decomposition of PAN, thus reducing the heat-release rate and interrupting the radical reaction during combustion. In the solid phase, the char generated from ADEP inhibits combustion by protecting the polymer surface from fire as well as isolating it from the air [27,28]. To evaluate the effect of ADEP on the flame retardancy of the composites, a vertical burning test, similar to UL94-VTM (vertical thin material) [29], was conducted, and varying contents of ADEP (0–20 wt%) were used. Generally, two-step ignitions (3 s each) are applied to the specimen for UL94-VTM; however, one-step ignition was applied in this work. For the electrochemical properties, ionic conductivity, rate capacity performance, and cycle stability were investigated using the PAN/ADEP composite membranes employed in the LIB coin cell. As a result, we demonstrated that the excellent heat-resistant and flame-retardant PAN/ADEP composite membranes can be used to solve the critical safety issues of LIBs.

## 2. Materials and Methods

### 2.1. Materials

PAN powder (copolymer of acrylonitrile (94 mol%) and methylacrylate (6 mol%), *M_w_* = 80,000) was purchased from Polysciences Inc., Warrington, PA, USA. *N*,*N*-dimethylformamide (DMF) and ADEP were supplied by Junsei Chemical Co., Ltd., Tokyo, Japan, and Universal Chemtech. Co., Ltd., Nonsan-si, Korea, respectively. Lithium nickel cobalt manganese oxide (NCM622, Ni:Co:Mn = 6:2:2, 7.7 mg/cm^2^), lithium metal, and 1 M lithium hexafluorophosphate (LiPF_6_) in ethylene carbonate (EC) and dimethyl carbonate (DMC) (1/1 (*v/v*)) with 10 wt% fluoroethylene carbonate (FEC) and 2 wt% vinylene carbonate (VC) were provided by Wellcos Co., Gunpo-si, Korea, and used as received. Celgard 2400 (~40% in porosity and 25 μm in thickness), supplied by Celgard LLC., Charlotte, NC, USA, was used as a reference separator.

### 2.2. Preparation of the PAN Composite-Based Porous Membranes

Firstly, the PAN powder was dissolved in DMF at a concentration of 15 wt% after drying it in a vacuum oven at 80 °C for 24 h. Subsequently, the polymer solution was placed into a 10 mL plastic syringe connected with a stainless-steel needle (MN-21G-13, Iwashita Engineering Inc., Kitakyushu, Japan) and electrospun at a feed rate of 1.2 mL/h and a voltage of 17 kV using a syringe pump (LEGATO100, KD Scientific Inc., Holliston, MA, USA) equipped with a high-voltage power supply (SHV200R, ConverTech Co. Ltd., Gwangmyeong-si, Korea). The relative humidity was set below 40%, and the distance between the syringe tip and the rotating drum collector was 17 cm. The electrospun PAN membrane detached from the collector was hot-pressed under 30 kgf/cm^2^ at 70 °C for 10 min, and then dried in a vacuum oven at 80 °C for 10 h to remove the residual solvent. The thickness of the membrane was fixed at 25 ± 2 μm. Finally, the heat treatment for the membranes under air at 210 °C for 30 min was conducted to improve the mechanical and thermal properties. For the composite membranes, the PAN powder was dissolved with varying contents of ADEP from 5 to 20 wt% in DMF, and thereafter the PAN/ADEP solution was electrospun under the same condition as the pristine membrane. Scheme 1 shows a schematic illustration of the preparation of the PAN/ADEP composite membranes by electrospinning.

### 2.3. Characterization of the Membranes

The nanofibrous morphologies of the membrane were examined using a scanning electron microscope (SEM, Vega II LSU, TESCAN, Brno, Czech Republic) after sputter coating (SC7640, Quorum Technologies Inc., Sacramento, CA, USA) with platinum under a vacuum for 2 min. The porosity of the membrane was determined from n-butanol uptake after immersing the membrane in n-butanol for 2 h, and it was calculated as follows:

(1) $$\mathrm{Porosity}\;\left(\%\right)=\frac{m_{b}-m_{d}}{\rho_{b}\times V}\times 100$$

where *m_b_* and *m_d_* are the weight of the n-butanol-filled and dry membranes, *ρ_b_* is the density of n-butanol, and *V* is the geometric volume of the dry membrane [30]. To characterize the pore size and pore size distribution of the membrane, an advanced capillary flow porometer (ACFP-1500AE, wet up/dry up mode, Porous Materials Inc., Ithaca, NY, USA) was used with Galwick solution. Thermalgravimetric analysis (TGA) was conducted with Pyris 1 TGA (PerkinElemer Inc., Waltham, MA, USA) at a heating rate of 10 °C/min under nitrogen gas from 30 to 800 °C. The mechanical strength of the membrane was measured using a material testing machine (LLOYD instrument, LR5K) with a cross-head speed of 10 mm/min under ambient conditions. The width and length of the specimen were 1 and 4 cm, respectively, and five specimens were tested to obtain the average values of the tensile strength and Young’s modulus for each membrane. The areal thermal shrinkage of the membrane was evaluated in an oven under air at 200 °C for 1 h, and it was calculated as follows:

(2) $$\mathrm{Areal}\;\mathrm{thermal}\;\mathrm{shrinkage}\;\left(\%\right)=\frac{A_{0}-A}{A_{0}}\times 100$$

where *A*_0_ and *A* are the area of the membrane before and after heat treatment, respectively. Contact angle measurements (PHX 300 mode) were performed on the membrane (a circle shape with a diameter of 18 mm) at 25 °C and 50% relative humidity using a static sessile drop mode. LE (1 M LiPF_6_ in EC/DMC (1/1 *v/v*)) was used as a liquid droplet to observe the wetting properties. The electrolyte uptake of the membrane was obtained from the weight difference between the dry and saturated membranes after soaking the membrane in the electrolyte for 1 h, and it was calculated as follows:

(3) $$\mathrm{Electrolyte}\;\mathrm{uptake}\;\left(\%\right)=\frac{M_{e}-M_{d}}{M_{d}}\times 100$$

where *M_e_* and *M_d_* are the weights of the membrane before and after immersion in the electrolyte, respectively.

To examine the effect of ADEP on the flame retardancy, the vertical burning test of the PAN/ADEP composite films was conducted. The width, length, and thickness of the specimen were 13 mm, 125 mm, and 60 μm, respectively, and five specimens were tested to obtain the average value of each film. The films were ignited for 3 s, and thereafter, the burning time was recorded as a function of the content of ADEP. Additionally, the flammability of the membrane prepared in a circle shape with a diameter of 18 mm was evaluated after immersion in the electrolyte and compared to that of the commercial PP membrane. The membranes were ignited for 0.5 s.

### 2.4. Electrochemical Evaluation of the Membranes

To determine the ionic conductivity of the membrane, the bulk impedance of the coin cell (CR2032 type) was analyzed by electrochemical impedance spectroscopy (EIS, SP-300, Biologic Science Instruments Ltd., Seyssinet-Pariset, France). The coin cell was assembled with the membrane sandwiched between two stainless-steel (SS) plates and filled with LE of 300 μL (SS/membrane with LE/SS cell). With an AC amplitude of 10 mV, the impedance spectra of the coin cells were recorded in a frequency range of 0.1 Hz–5 MHz at room temperature. The ionic conductivity (σ) was calculated as follows:

(4) $$\sigma \;\left(mS/cm\right)=\frac{d}{R_{b}\times A}$$

where *d* and *A* are the thickness and effective area of the membrane, and *R_b_* is the bulk impedance given by the *x*-intercept on the EIS graph.

For the electrochemical stability of the membrane, the coin cell was assembled with the membrane sandwiched between lithium metal and an SS plate and filled with LE of 300 μL (Li/membrane with LE/SS cell). Subsequently, the linear sweep voltammetry (LSV) measurement was performed using the coin cell at a scan rate of 0.1 mV/s in the range of 3.0–5.7 V under ambient conditions. To examine the heat resistance of the membrane, the LE-soaked membrane was sandwiched between two SS plates with Kapton tape and then heated from 30 to 200 °C in a temperature-controlled oven (DZF-6020-FP, MTI Corporation, Richmond, CA, USA) at a rate of 2 °C/min. Using an electrochemical workstation (ZIVE MP2, WonATech Co. Ltd., Seoul, Korea), the electrochemical impedance of the assembled sample was continuously measured at 1 kHz.

The battery performance of the membrane was evaluated using the coin cell (half-cell) that was assembled with the membrane sandwiched between the lithium metal (anode) and NCM622 (cathode) and filled with LE of 300 μL (Li/membrane with LE/NCM622 cell). The interfacial resistance of the coin cell between the LE-soaked membrane and electrodes was obtained from the EIS measured in a frequency range of 0.1 Hz–5 MHz. Additionally, the discharge C-rate capability was examined at varying current densities from 0.2 (0.26 mA/cm^2^) to 5 C, and the cyclic performance was recorded at a fixed current density of 1 C using an automatic battery test system (WBCS3000L, WonATech Co. Ltd., Seoul, Korea) in a voltage range of 3.0–4.2 V at room temperature.

## 3. Results and Discussion

### 3.1. Characterization of the PAN/ADEP Composite Membranes

**Figure 1:**
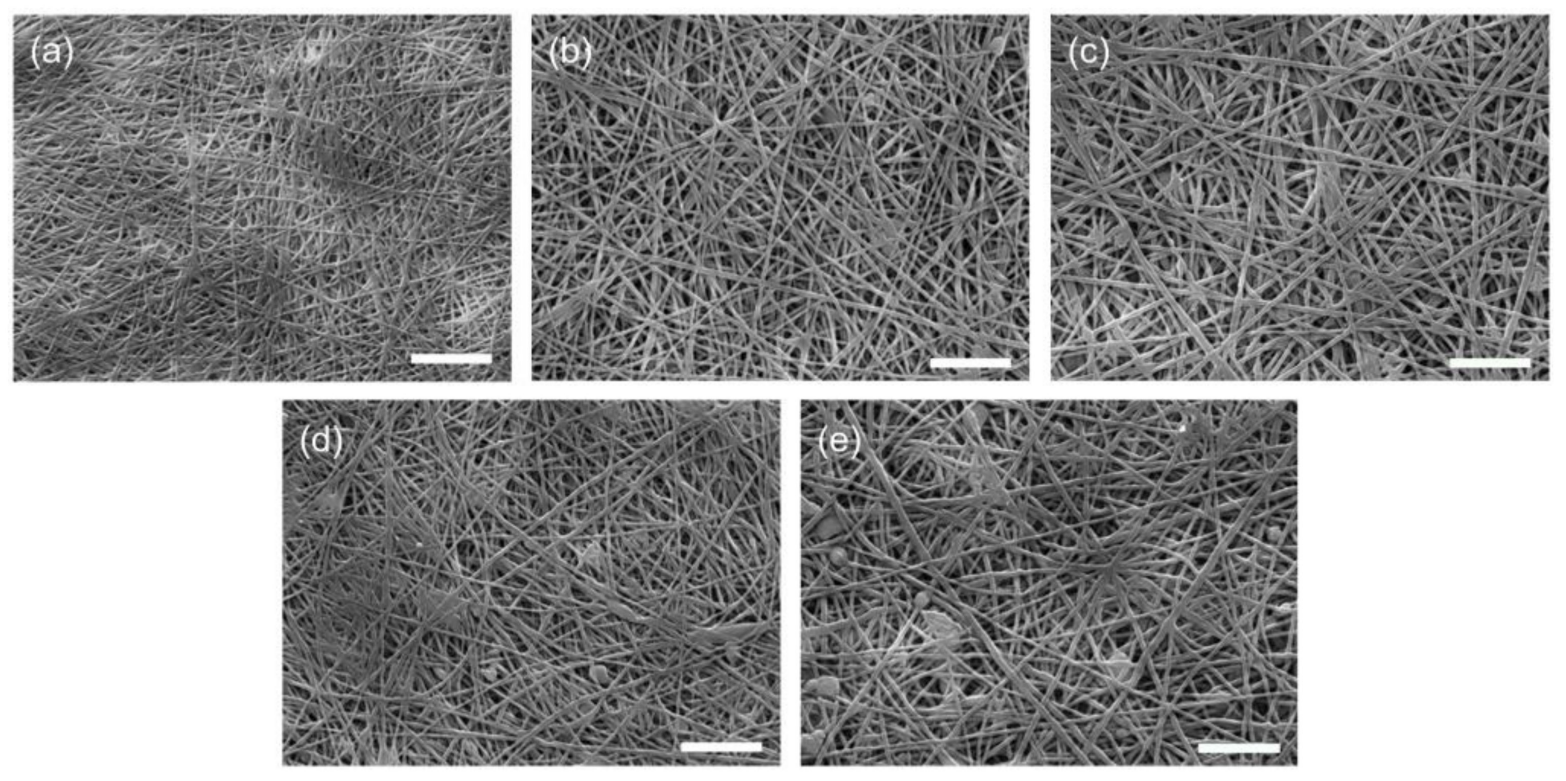
Surface SEM images of the PAN/ADEP composite membranes after sputter coating with platinum under a vacuum for 2 min: (**a**) PAN; (**b**) PAN/AD5; (**c**) PAN/AD10; (**d**) PAN/AD15; (**e**) PAN/AD20. The scale bars represent 10 μm.

The nanoporous PAN/ADEP composite membranes were prepared with varying contents of ADEP from 5 to 20 wt% and heat-treated for partial oxidation at 210 °C for 30 min. The oxidation reaction of PAN changed the linear structure of the molecular chain to cyclic, aromatic, and ladder structures, improving the mechanical and thermal properties [31]. The composite membranes are denoted by the different amounts of ADEP as PAN/ADX, where X is the weight percent of ADEP. The flame-retardant ADEP is a highly thermally stable phosphorus compound with its thermal degradation beginning at ~390 °C. This indicated that ADEP did not degrade during the oxidation of the PAN membranes (Figure S1). The surface morphologies of the PAN and PAN/ADEP composite membranes are shown in Figure 1. Regarding the PAN membrane, the average fiber diameter and pore size were 0.31 and 0.22 μm, respectively, and the nanofibers were randomly aligned without microscopically identified beads that could negatively affect the physical properties of the membrane. In addition, no evident deformations of the membranes were observed after hot-pressing at 70 °C. However, the composite membranes showed a higher fiber diameter (0.48–0.50 μm) and pore size (0.37–0.38 μm) than those of the PAN membrane (see Figure S2 for the pore size distributions). This was because ADEP increased the viscosity of the polymer solution, which is the same as the effect of an increase in the concentration of the polymer [18]. When the content of ADEP was more than 10 wt%, small beads and beaded nanofibers were observed on the surface of the membranes, leading to a decrease in the porosity to less than 45% due to the aggregation of ADEP.

**Figure 2:**
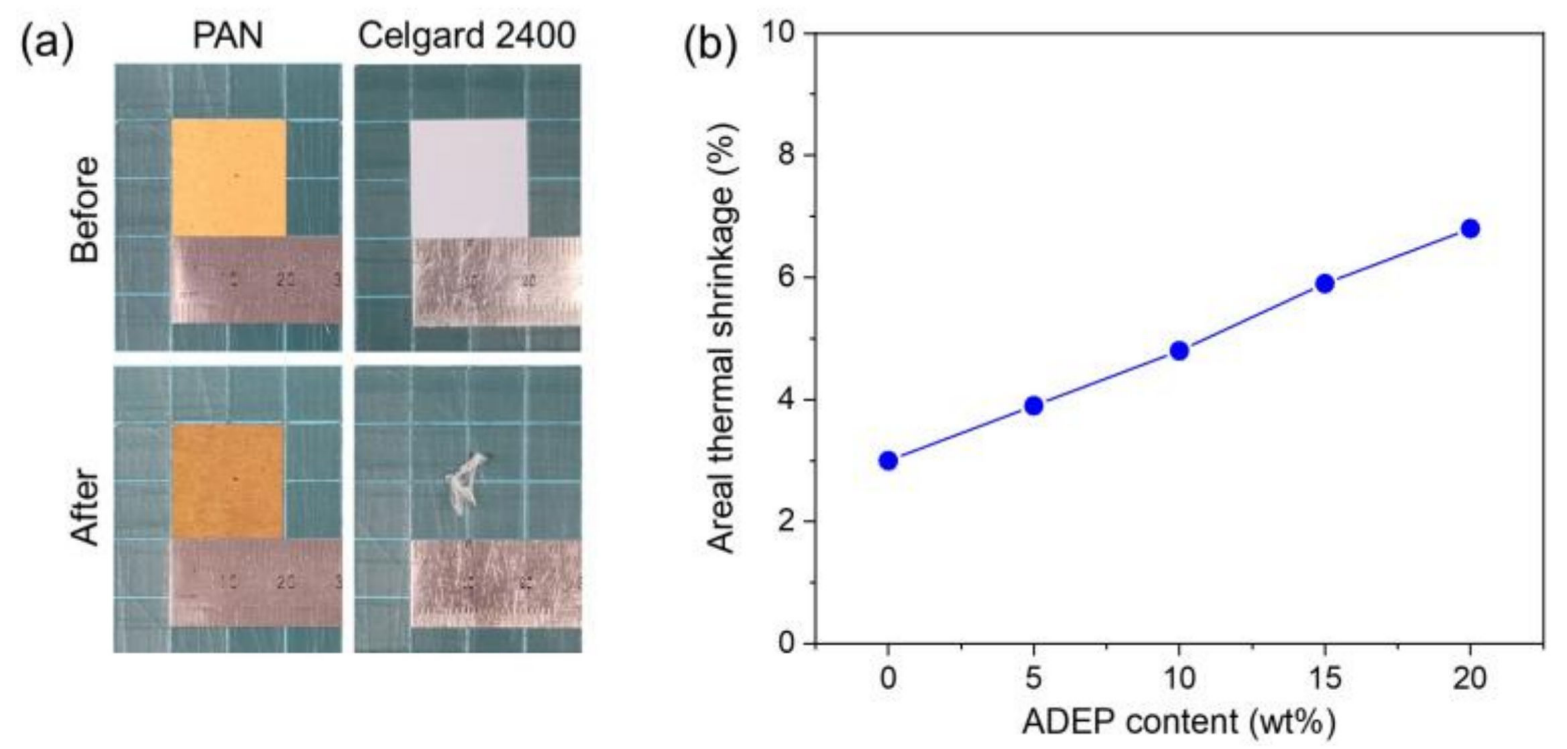
(**a**) Digital photographs of the PAN and Celgard 2400 membranes before and after thermal exposure in air at 200 °C for 1 h; (**b**) the areal thermal shrinkages of the PAN membranes as a function of the content of ADEP.

The mechanical properties of the PAN/ADEP composite membranes were tested under ambient conditions. With an increase in the content of ADEP, the tensile strength and Young’s modulus decreased because flame retardants generally decrease the mechanical properties when they are incorporated because of their monomeric or oligomeric structures. Therefore, ADEP adversely affected the tensile strength and Young’s modulus of the membranes in contrast to the rigid inorganic materials. However, the tensile strengths and Young’s moduli of the composite membranes were maintained over 25 MPa and 1.0 GPa (27.7 MPa and 1.1 GPa for PAN/AD10), respectively, when the content of ADEP was less than 15 wt%. PAN/AD15 and PAN/AD20 showed a further decrease in their mechanical properties because of the defects that originated from the aggregation of numerous organic compounds. Table 1 summarized the porosity, pore size, fiber diameter, and mechanical properties of the membranes as a function of the content of ADEP. For the areal thermal shrinkage, the PAN and composite membranes maintained their dimensional shape without obvious shrinkage after thermal exposure at 200 °C for 1 h, whereas Celgard 2400, a porous PP membrane, shrank extensively and melted down, as shown in Figure 2a and Figure S3. With an increase in the content of ADEP, the areal thermal shrinkage of the composite membrane increased from 3.0% to 6.8%; however, it was still less than 10%, indicating that the negative effect of ADEP on the areal thermal stability was quite small, because ADEP was also highly stable at ≤390 °C (Figure 2b). As a result, the thermally stable composite membranes could effectively prevent internal short-circuit failures when the temperature increased to >160 °C.

### 3.2. Flame Retardancy of the Membranes

**Figure 3:**
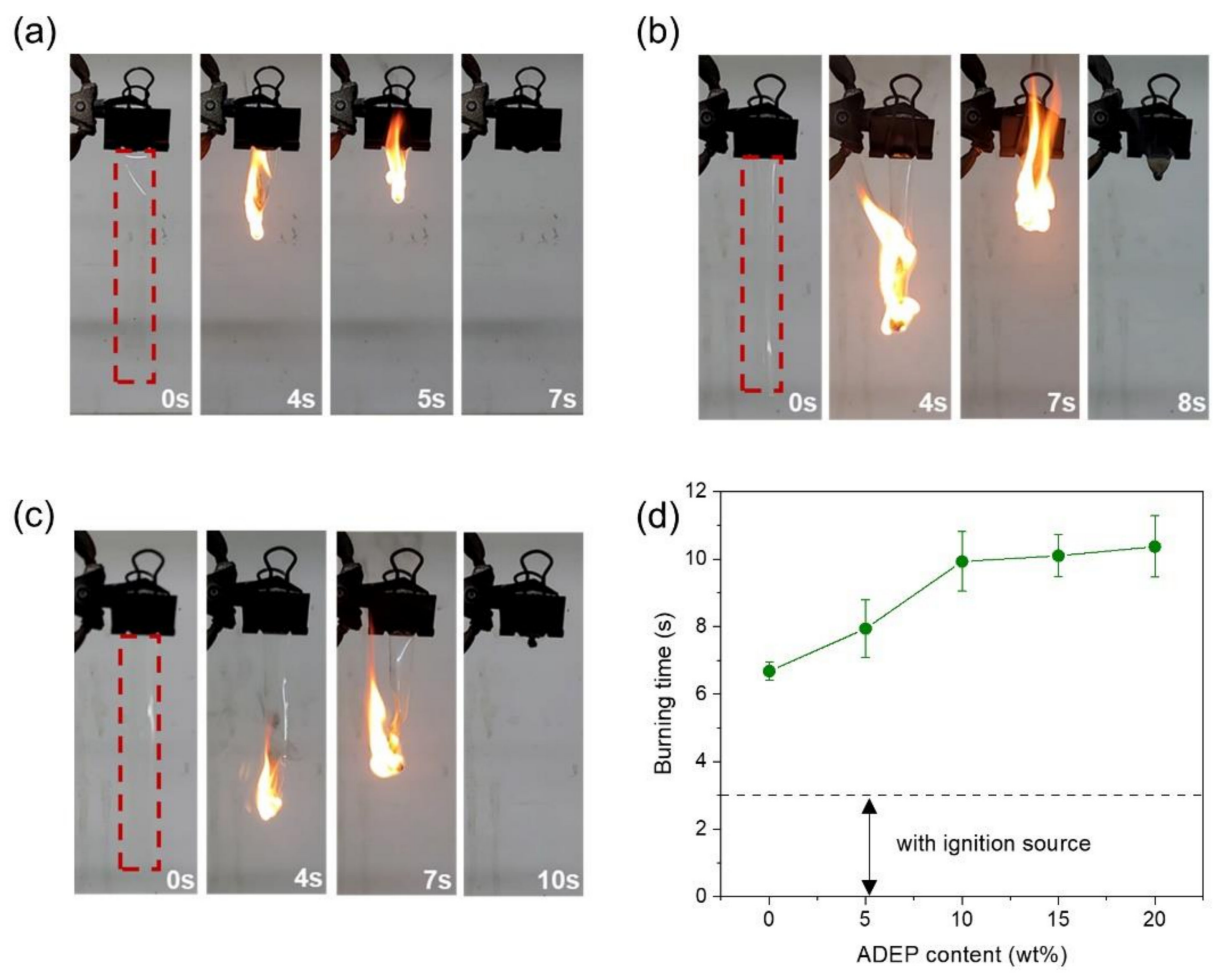
Digital photographs of the vertical burning test for (**a**) the pristine PAN film and the composite films containing (**b**) 5 wt% and (**c**) 10 wt% ADEP. The films were ignited for 3 s and (**d**) the burning time as a function of the content of ADEP was recorded.

The flame retardancy of the membranes is important to overcome the safety issues of LIBs, since polyolefin membranes are easily burned when the flame is generated [32,33]. To evaluate the effect of ADEP on the flame retardancy of the PAN/ADEP composites, a vertical burning test was conducted under ambient conditions. The samples were prepared as a film (13 mm (width) × 125 mm (length) × 60 μm (thickness)) and ignited for 3 s to perform the test. In Figure 3a, photographs of the vertical burning test for the pristine PAN film are presented according to the burning time. After exposure to the flame for 3 s, the PAN film was continuously burned up for ~4 s until it completely disappeared. When the contents of ADEP were 5 and 10 wt% in the composite films, the burning time increased to ~5 and ~7 s, respectively, because ADEP suppressed the burning speed of the films (Figure 3b,c). For the composite films containing 15 and 20 wt% ADEP, the burning time increased slightly as shown in Figure 3d and Figure S4. These results demonstrated that the flame retardancy was effectively improved by ADEP when more than 5 wt% was introduced into PAN. Considering the mechanical and thermal properties and the nonflammability, the optimum content of ADEP for the PAN composite was found to be 10 wt% in this study.

**Figure 4:**
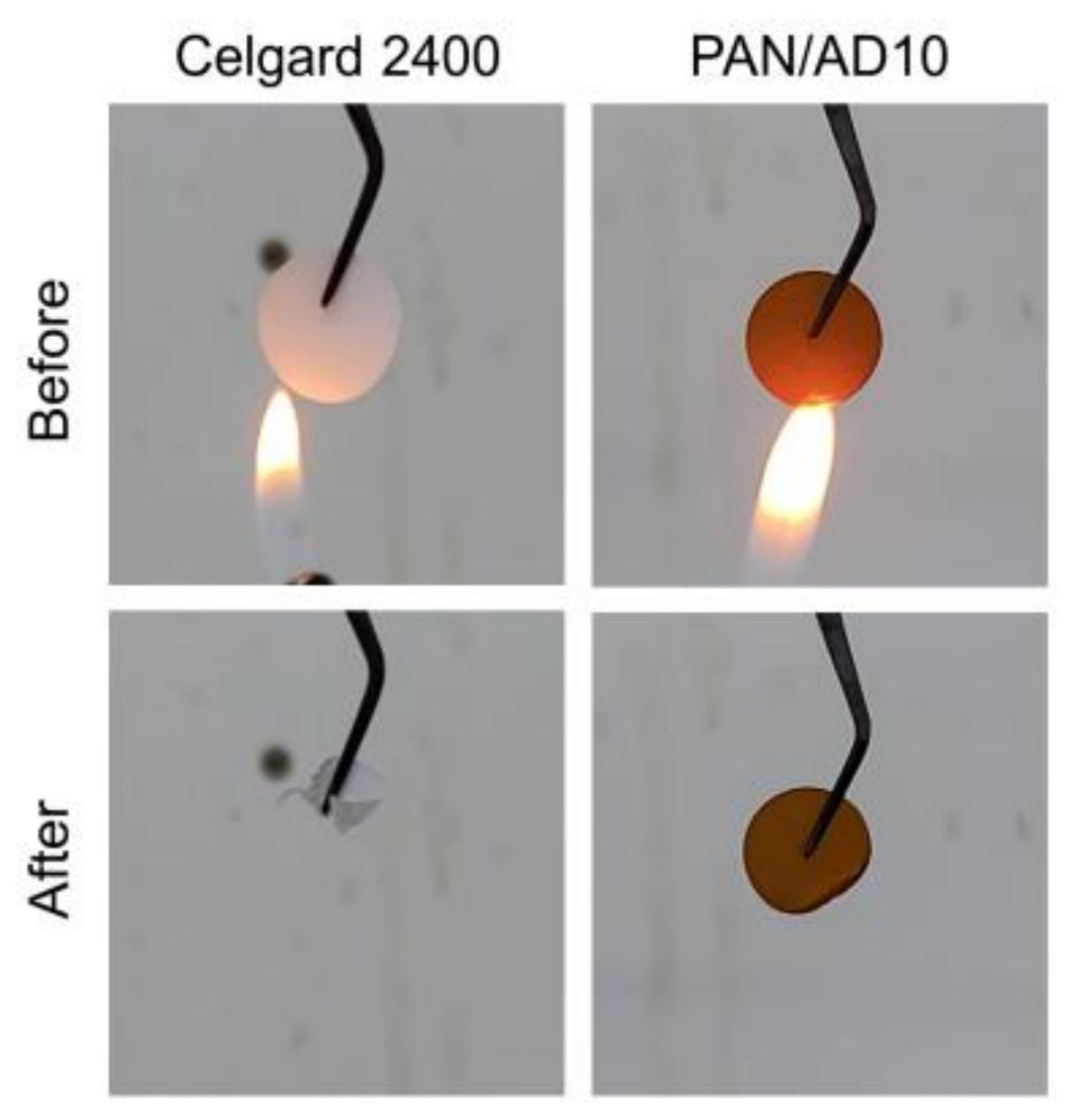
Digital photographs of the burning test for Celgard 2400 and PAN/AD10. The membranes were completely wet with LE (1 M LiPF_6_ in EC/DMC (1/1 *v/v*)).

Additionally, the flame retardancy of the membrane as a separator was examined using a circular specimen with a diameter of 18 mm after immersion in LE for 1 h. Figure 4 shows the burning test for Celgard 2400 and PAN/AD10 before and after exposure to the flame. When the flame was closed, Celgard 2400 shank extensively and immediately melted, while the circular shape of PAN/AD10 was maintained without significant deformations, identifying its good nonflammability. From these results, it was confirmed that the heat-resistant and flame-retardant PAN/AD10 membrane is a promising candidate for enhancing the safety of LIBs.

### 3.3. Electrolyte Wettability of the Membranes

**Figure 5:**
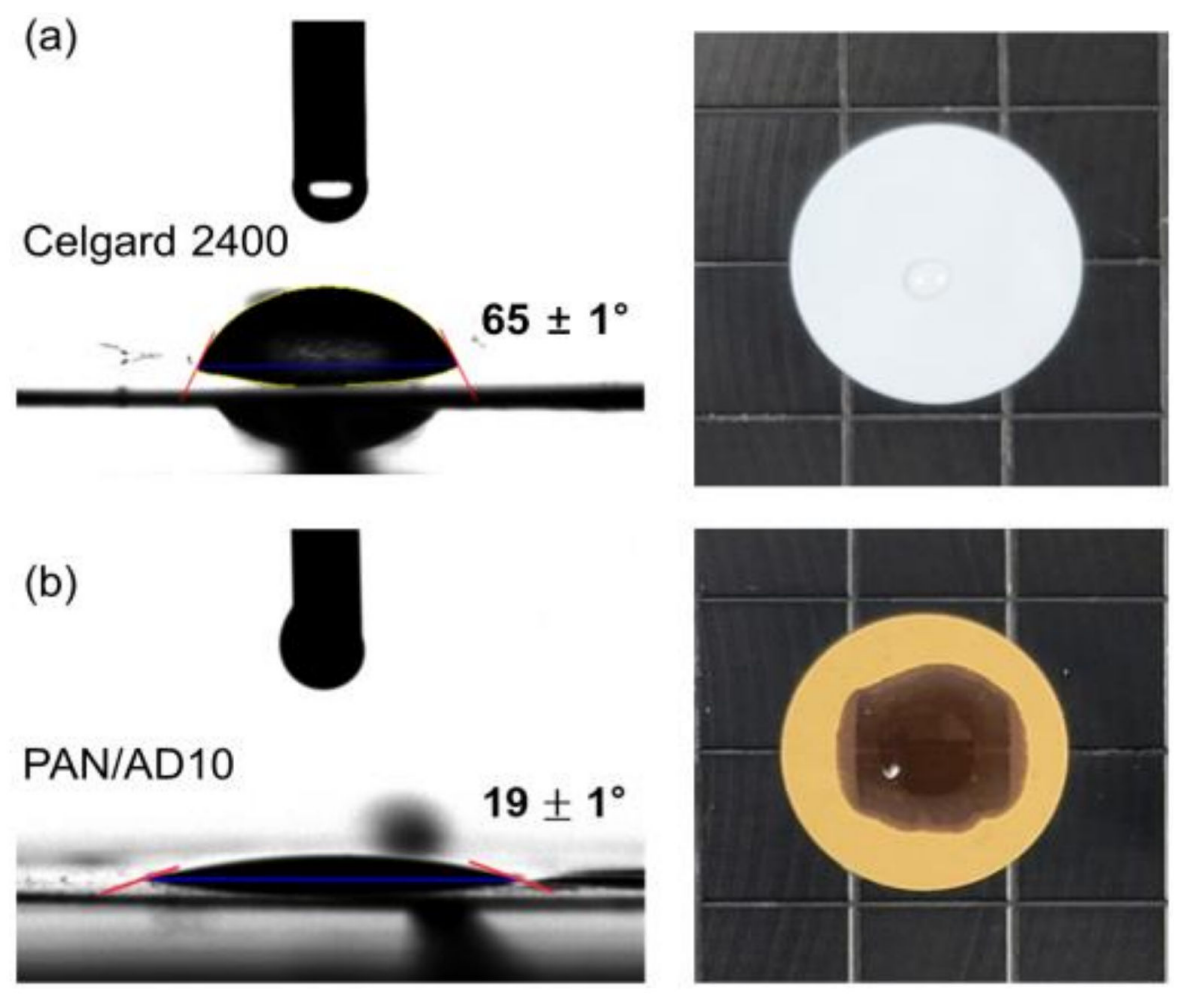
Images of the contact angle and wettability between the membrane and LE: (**a**) Celgard 2400 and (**b**) PAN/AD10.

To improve lithium-ion transfer, good wettability is essential for filling LE into the pores of the membranes [19,34]. Contact angle measurements were performed at 25 °C and 50% relative humidity to examine the LE wettability of the membrane. Figure 5 shows the images of the contact angles and surface wettability between the membranes, Celgard 2400 and PAN/AD10, and LE. Because of the hydrophobic surface of the polyolefin membrane, Celgard 2400 was not wet when LE was dropped onto its surface; however, the surface of PAN/AD10 was rapidly wetted by an LE droplet that easily spread over a wide area of the membrane due to the hydrophilic nature of its nitrile groups. The contact angle of the LE droplet on PAN/AD10 was found to be 19°, which is considerably lower than that on Celgard 2400 (65°), resulting in superior electrolyte uptake and lithium-ion transport. The electrolyte uptake values of Celgard 2400 and PAN/AD10 were calculated from the weight differences between the dry and saturated membranes after soaking the membranes in LE for 1 h. As expected, the electrolyte uptake of PAN/AD10 was found to be 137%, which was higher than that of Celgard 2400 (63%). To characterize the ion conductivity, the EIS measurements were performed using the coin cells assembled with the membrane and two SS plates (SS/membrane with LE/SS cell) under ambient conditions. The bulk resistance (*R_b_*) was determined from the *x*-intercept on an EIS graph, as shown in Figure S5, and the same correlation with the electrolyte uptake was observed in the ionic conductivity because of the hydrophilic nature of PAN. The ionic conductivities of Celgard 2400 and PAN/AD10 were found to be 0.69 and 0.83 mS/cm, respectively, indicating that the hydrophilic chemical composition and interconnected pore structure can improve lithium-ion transport through the membrane for high-performance LIBs.

### 3.4. Electrochemical Stability and Heat Resistance of the Membranes

**Figure 6:**
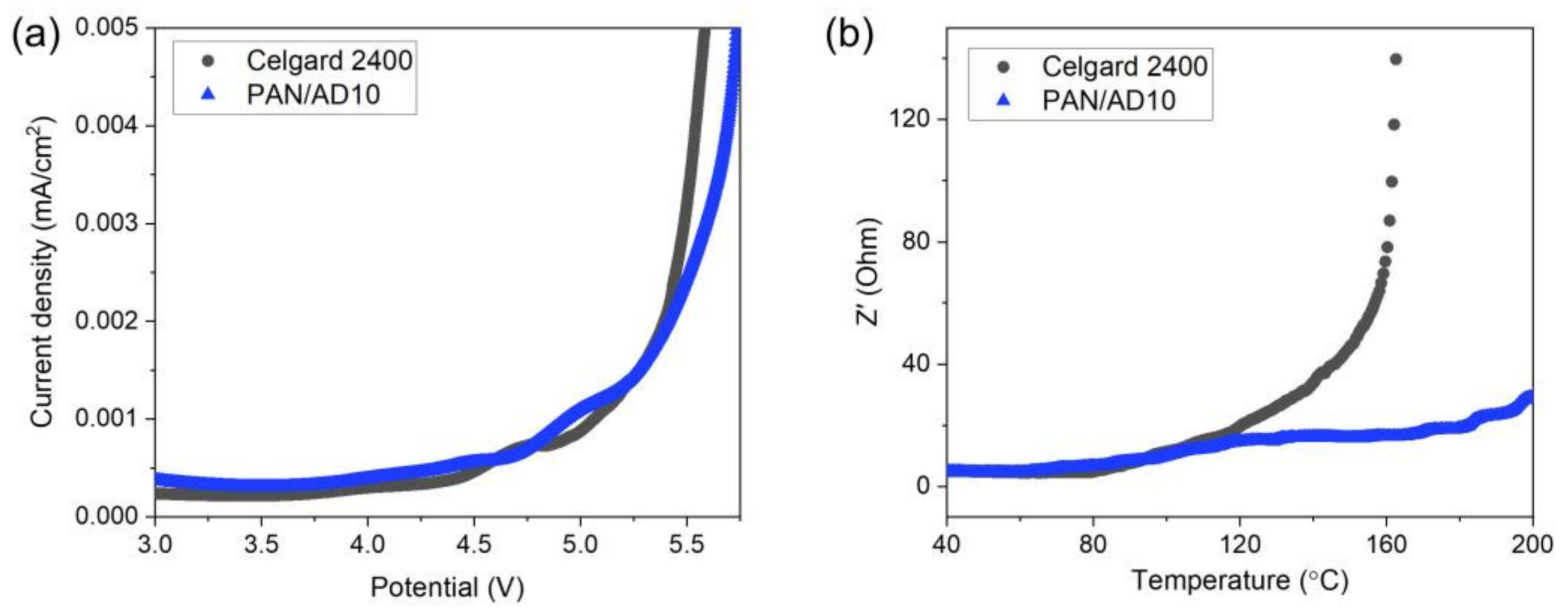
(**a**) Electrochemical stability and (**b**) heat resistance curves of Celgard 2400 and PAN/AD10 with LE. The electrochemical stability (Li/membranes with LE/SS cells) was measured at room temperature and a scan rate of 0.1 mV/s in the range of 3.0−5.7 V. For the heat resistance (SS/membrane with LE/SS with Kapton tape), the cells were heated to 200 °C at a rate of 2 °C/min, and their electrochemical impedances were simultaneously measured at 1 kHz.

For the electrochemical stability, the LSV measurements of the coin cells assembled with the membrane, lithium metal, and SS plate (Li/membrane with LE/SS cell) were performed under ambient conditions. Figure 6a shows the electrochemical stabilities of Celgard 2400 and PAN/AD10 in the range of 3.0–5.7 V under ambient conditions. The LE-soaked PAN/AD10 membrane was highly stable without decomposition of any components under 4.5 V vs. Li + /Li, which was similar to the Celgard 2400 membrane [35,36]. It verified that the composite membrane with LE had good electrochemical stability under LIB operating conditions. The heat resistances of the membranes were investigated by measuring the impedance of the LE-soaked membrane sandwiched between two SS plates and sealed with Kapton tape at elevated temperatures. In a temperature-controlled oven, the assembled sample was heated to 200 °C, and the EIS was simultaneously analyzed at 1 kHz. Figure 6b shows the impedance variations of Celgard 2400 and PAN/AD10 in the temperature range of 40–200 °C. Since the PP membrane shrank extensively and melted at an elevated temperature, the impedance of the Celgard 2400 cell drastically increased above 160 °C; however, the impedance of the PAN/AD10 cell was highly stable, even up to 200 °C, owing to its outstanding thermal dimensional stability. From these results, it was confirmed that the composite membrane can endure thermal deformations over a wide range of temperatures for safe LIBs.

### 3.5. Electrochemical Performance of the Membranes

The battery performance of the membranes was analyzed using the coin cells prepared with the LE-soaked membrane sandwiched between the lithium metal (anode) and NCM622 (cathode) (Li/membrane with LE/NCM622 cell). To investigate the interfacial compatibility between the LE and electrodes in the coin cells of Celgard 2400 and PAN/AD10, EIS measurements were conducted in the range of 0.1 Hz–5 MHz under ambient conditions as shown in Figure S6a. The EIS curves show a depressed semicircle in the high-frequency region and a sloped line in the low-frequency region, which indicates the charge transfer reactions at the electrolyte–electrode interface and Warburg impedance on the diffusion of Li+ in the NCM particles, respectively [24,30,32]. Figure S6b shows the equivalent circuit model to fit the EIS curves; *R_s_* is the ohmic resistance of the cell including the resistances of the membrane, electrolyte, and electrodes; *R_ct_* is the charge transfer resistance; CPE is the double-layer capacitance and passivation film capacitance; *Z_w_* indicates the Warburg impedance. The *R_ct_* value of PAN/AD10 (112 Ω) was lower than that of Celgard 2400 (154 Ω), arising from the improved lithium-ion transport through the PAN/AD10 membrane compared to that through the Celgard 2400 membrane. Moreover, the higher electrolyte uptake capacity of PAN/AD10 facilitated superior interfacial contact with the electrodes.

**Figure 7:**
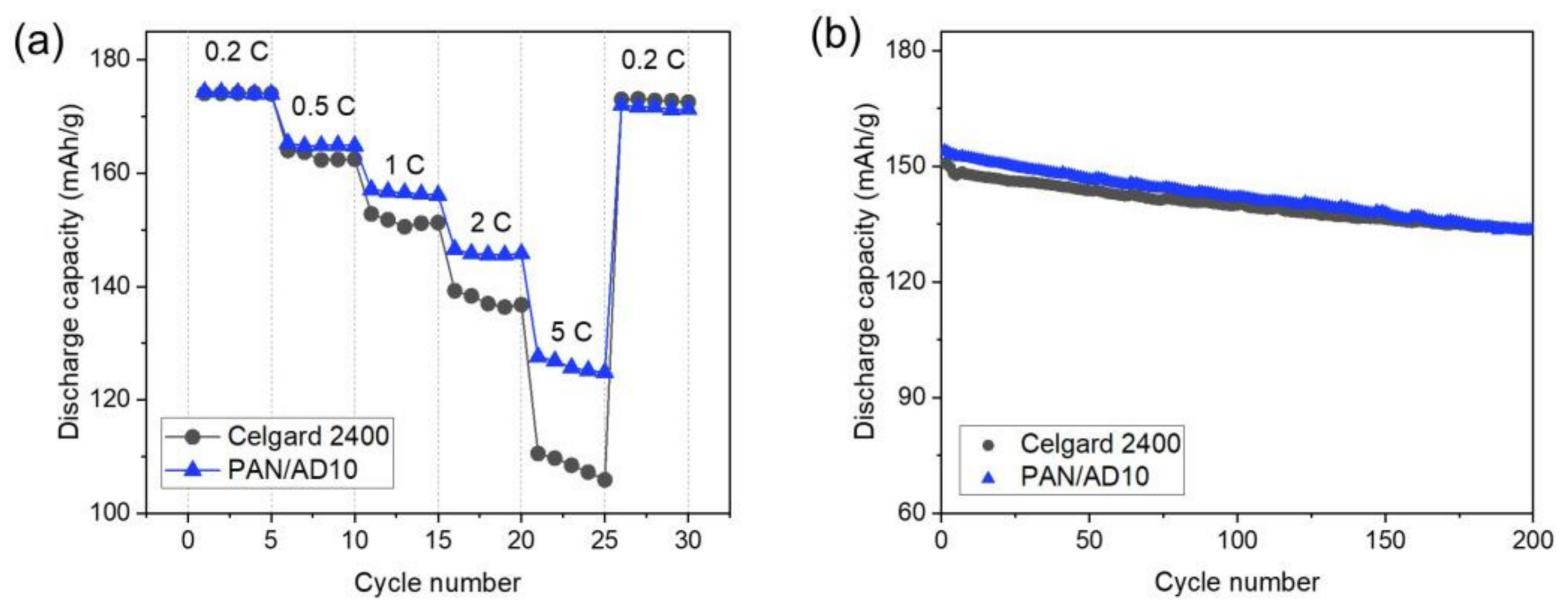
Comparison of (**a**) the discharge C-rate capabilities and (**b**) cycle performances (1.0 C-rate) between Celgard 2400 and PAN/AD10 (Li/membrane with LE/NCM622 cells, 3.0−4.2 V, room temperature).

In Figure 7a, the galvanostatic rate performances of the coin cells of Celgard 2400 and PAN/AD10 are presented in the voltage range of 3.0–4.2 V at five different C-rates (0.2, 0.5, 1, 2, and 5 C). At a C-rate of 0.2 C, the highest discharge capacities of the Celgard 2400- and PAN/AD10-based cells were found to both be 174 mAh/g. The discharge capacities of the coin cells decreased with an increase in the C-rate, because the diffusion phenomena occurred within the electrode active material phase and the LE-soaked membranes. The discharge capacities of PAN/AD10 at 1, 2, and 5 C were found to be 157, 146, and 126 mAh/g, respectively, which are the enhanced values compared to those of Celgard 2400 (152, 138, and 110 mAh/g, respectively), arising from the higher ionic conductivity and lower interfacial resistance of the PAN/AD10-based cell. The cycling stabilities of the Celgard 2400- and PAN/AD10-based cells were also evaluated at a C-rate of 1 C for up to 200 cycles as shown in Figure 7b. The cycle performance of the PAN/AD10-based cell was considerably stable, and the capacity retention was found to be 87%, which was comparable to that of the Celgard 2400-based cell (88%) (see Figure S7 for the charge–discharge curves). In addition, the discharge capacities of Celgard 2400 and PAN/AD10 after 200 cycles were the same, implying that the developed composite membrane showed sufficient electrochemical performances for use in safe LIBs.

## 4. Conclusions

Electrospun PAN/ADEP porous composite membranes were developed by incorporating a flame-retardant ADEP, varying from 0 to 20 wt%, for safety of LIBs. To improve the mechanical and thermal properties, the PAN/ADEP composite membranes were partially oxidized by thermal exposure at 210 °C for 30 min. The flame resistance of the composite membranes was examined by a burning test and effectively enhanced by ADEP when more than 5 wt% to PAN was added. The optimum content of ADEP was found to be 10 wt% for the heat resistance and flame retardancy of the membrane. Additionally, the electrolyte uptake and ion conductivity of PAN/AD10 (137% and 0.83 mS/cm) were superior to those of Celgard 2400 (63% and 0.65 mS/cm), since PAN/AD10 has interconnected pores and a hydrophilic chemical composition. The PAN/AD10-based cell showed excellent heat resistance as well as superior discharge capacities to those of the Celgard 2400-based cell at all C-rates because of the lower interfacial resistances. During 200 cycles, the capacity retention of PAN/AD10 at 1 C was also highly stable. Consequently, it was confirmed that the PAN/ADEP composite membrane can be used as a nonflammable separator for high-performance and safe LIBs.

## Figures and Tables

**Table 1:** The fiber diameter, pore size, porosity, and mechanical properties of the PAN membranes as a function of the content of ADEP.

Sample	ADEP Content (%)	Fiber Size (μm)	Average Pore Size (μm)	Porosity (%)	Tensile Strength (MPa)	Young’s Modulus (GPa)
PAN	0	0.31 ± 0.05	0.22	46 ± 2	47.2 ± 1.4	1.62 ± 0.05
PAN/AD5	5	0.48 ± 0.08	0.37	51 ± 2	28.9 ± 0.5	1.14 ± 0.02
PAN/AD10	10	0.49 ± 0.08	0.38	46 ± 2	27.7 ± 0.7	1.14 ± 0.03
PAN/AD15	15	0.49 ± 0.09	0.37	41 ± 2	23.4 ± 0.4	0.91 ± 0.02
PAN/AD20	20	0.50 ± 0.10	0.38	38 ± 2	19.4 ± 0.5	0.81 ± 0.03

## Data Availability

Not applicable.

## Supplementary Materials

The following supporting information can be downloaded at: https://www.mdpi.com/article/10.3390/polym14091649/s1, Figure S1: TGA curves for PAN, ADEP, and PAN/AD10; Figure S2: Pore size distribution of (a) the PAN; (b) PAN/AD5; (c) PAN/AD10; (d) PAN/AD15; (e) PAN/AD20 membranes; Figure S3: Digital photographs of the PAN/AD5, PAN/AD10, PAN/AD15, and PAN/AD20 membranes before and after thermal exposure in air at 200 °C for 1 h; Figure S4: Digital photographs of the vertical burning test for the composite film: (a) 15 wt% and (b) 20 wt% ADEP; Figure S5: Electrochemical impedance curves of Celgard 2400 and PAN/AD10 with the LE in the range of 0.1 Hz–5 MHz; Figure S6: (a) Electrochemical impedance curves of Celgard 2400 and PAN/AD10 in the range of 0.1 Hz–5 MHz and (b) equivalent circuit to fit the impedance curve; Figure S7: Charge–discharge curves at the 1st, 50th, 100th, and 200th cycles of (a) Celgard 2400 and (b) PAN/AD10.
