# Supplementary material for: Aluminum Diethylphosphinate-Incorporated Flame-Retardant Polyacrylonitrile Separators for Safety of Lithium-Ion Batteries
Source: Polymers (Basel). 2022 Apr 19;14(9):1649. doi: 10.3390/polym14091649 (PMC9100846; doi:10.3390/polym14091649)
Supplement: Supplementary file 1 [file polymers-14-01649-s001.zip › polymers-1669251-supplementary.pdf]

polymers

Supplementary Materials

Aluminum Diethylphosphinate-Incorporated Flame-Retardant Polyacrylonitrile Separators for Safety of Lithium-Ion Batteries

Seok Hyeon Kang [1,2,†], Hwan Yeop Jeong [1,†], Tae-Ho Kim [1], Jang Yong Lee [1], Sung-Kwon Hong [2], Young Taik Hong [1], Jaewon Choi [3], Soonyong So [1,*], Sang Jun Yoon [1,*] and Duk Man Yu [1,*]

1 Energy Materials Research Center, Korea Research Institute of Chemical Technology (KRICT), Daejeon 34114, Republic of Korea; mlc1207@krict.re.kr (S.H.K.); hwanyeop@krict.re.kr (H.Y.J.); thkim@krict.re.kr (T.-H.K.); ljylee@krict.re.kr (J.Y.L.); ythong@krict.re.kr (Y.T.H.)
2 Department of Polymer Engineering, Chungnam National University, Daejeon 34134, Republic of Korea; skhong@cnu.ac.kr (S.-K.H.)
3 Department of Polymer Science and Engineering, Kyungpook National University, Daegu, 41566, Republic of Korea; jwchoi@knu.ac.kr (J.C.)
* Correspondence: syso@krict.re.kr (S.S.); sjyoon@krict.re.kr (S.J.Y.); dmyu@krict.re.kr (D.M.Y.)
† These authors contributed equally to this work.

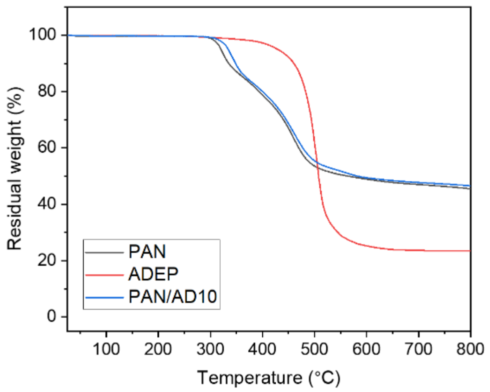

Figure S1. TGA curves for PAN, ADEP, and PAN/AD10. The samples were measured at a heating rate of 10 °C/min under nitrogen gas from 30 °C to 800 °C. The pristine and composite samples showed that the thermal degradation began at ~300 °C and finished at ~500 °C. Moreover, the thermal degradation of the flame retardant, ADEP, was occurred at ~390 °C, indicating that ADEP is highly stable during the oxidation of PAN.

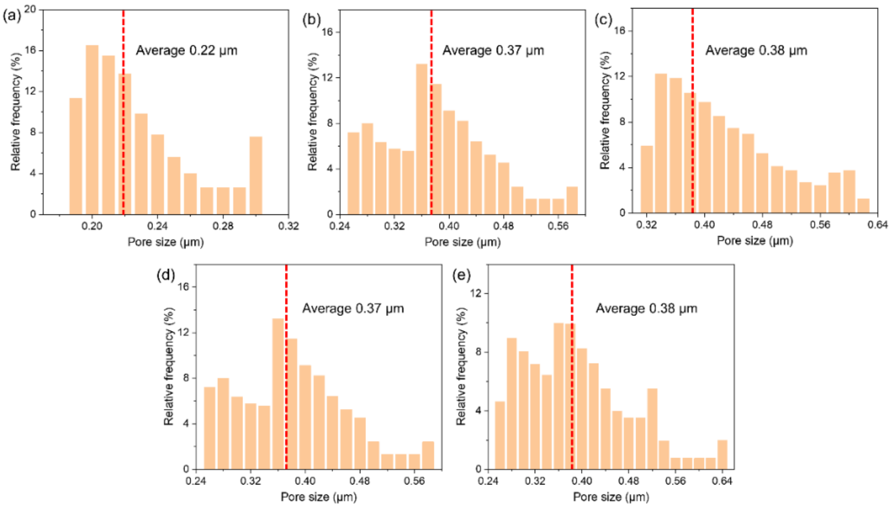

Figure S2. Pore size distribution of (**a**) the PAN, (**b**) PAN/AD5, (**c**) PAN/AD10, (d) PAN/AD15, and (e) PAN/AD20 membranes

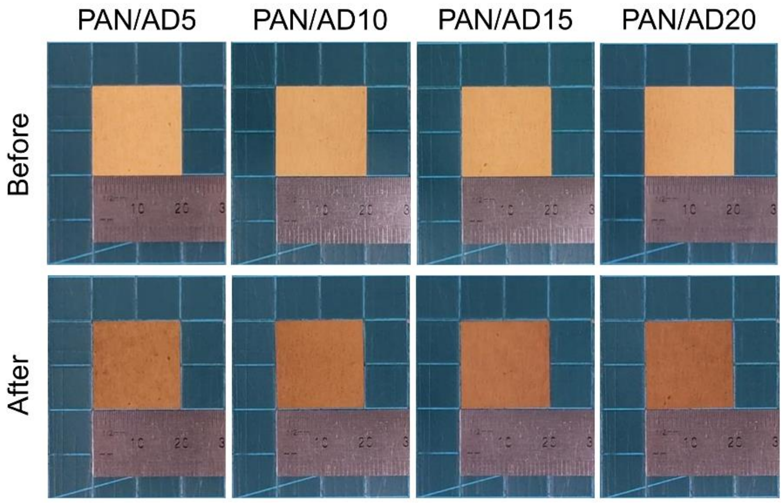

Figure S3. Digital photographs of the PAN/AD5, PAN/AD10, PAN/AD15, and PAN/AD20 membranes before and after thermal exposure in the air at 200 °C for 1 h.

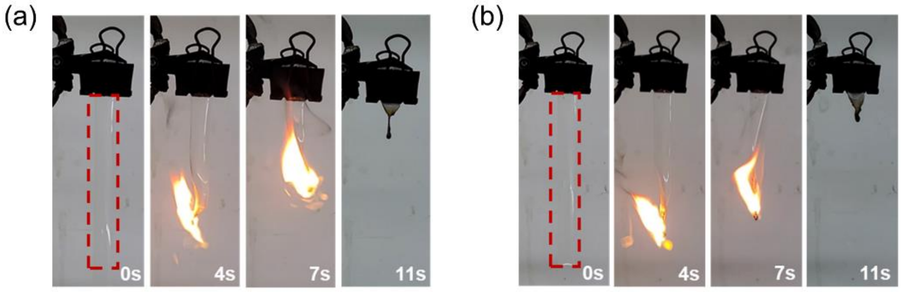

Figure S4. Digital photographs of the vertical burning test for the composite film; (**a**) 15 wt% and (**b**) 20 wt% ADEP. The films were ignited for 3 s.

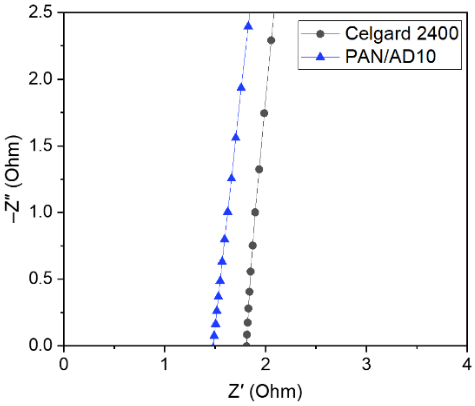

Figure S5. Electrochemical impedance curves of Celgard 2400 and PAN/AD10 with the LE (SS/membrane with LE/SS cells) in the range of 0.1 Hz–5 MHz.

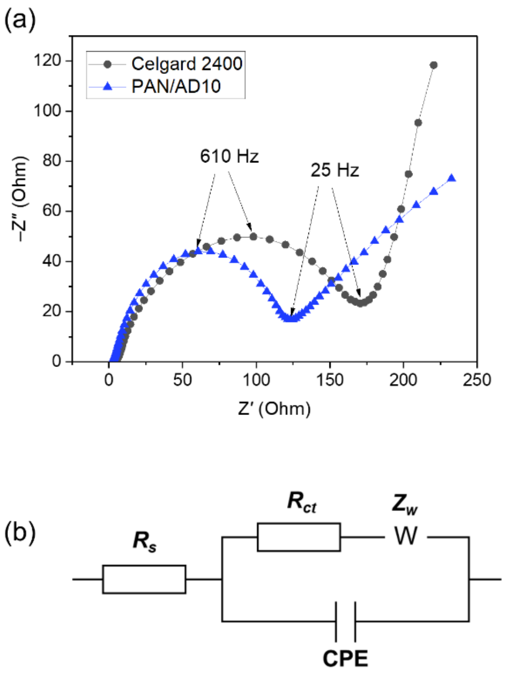

Figure S6. (**a**) Electrochemical impedance curves of Celgard 2400 and PAN/AD10 (Li/membrane with LE/NCM622 cells) in the range of 0.1 Hz–5 MHz and (**b**) equivalent circuit to fit the impedance curve.

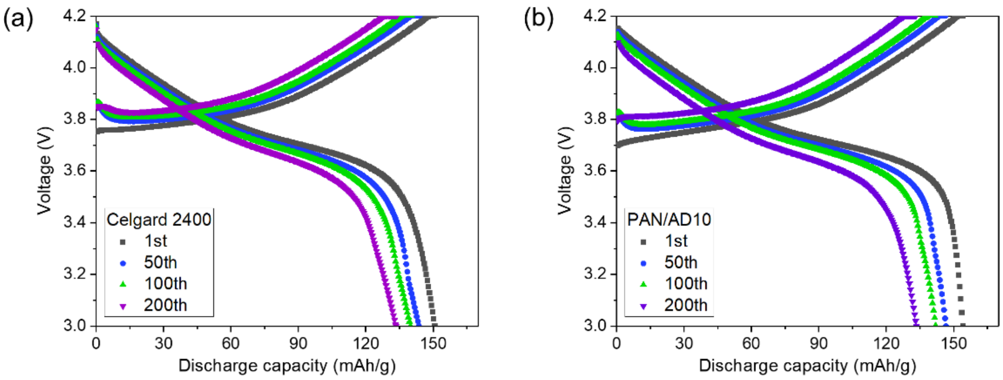

Figure S7. Charge-discharge curves at 1st, 50th, 100th, and 200th cycles of (**a**) Celgard 2400 and (**b**) PAN/AD10 (Li/membranes with LE/NCM622 cells, 3.0–4.2 V, room temperature).
